# Waist circumference and risk of Parkinson’s disease

**DOI:** 10.1038/s41531-022-00353-4

**Published:** 2022-07-08

**Authors:** Kye-Yeung Park, Ga Eun Nam, Kyungdo Han, Hoon-Ki Park, Hwan-Sik Hwang

**Affiliations:** 1 Department of Family Medicine, Hanyang University College of Medicine, Seoul, South Korea; 2 Department of Family Medicine, Korea University College of Medicine, Seoul, South Korea; 3 Department of Statistics and Actuarial Science, Soongsil University, Seoul, South Korea

**Keywords:** Risk factors, Epidemiology

## Abstract

Although many studies support the association of obesity with neurodegenerative diseases, such as Parkinson’s disease (PD), there are limited data regarding the association between abdominal obesity and PD, with mixed findings. The aim of this study was to examine the association of waist circumference (WC) with the risk of PD incidence. We retrospectively analyzed a large-scale nationwide cohort of 6,925,646 individuals aged ≥40 years who underwent the Korean National Health Screening during 2009. We performed multivariable Cox proportional hazards regression to evaluate the association of WC and abdominal obesity with PD risk and calculated hazard ratios (HRs) with 95% confidence intervals (CIs) of PD incidence. During a median follow-up period of 8.35 years, 33,300 cases of PD developed. PD incidence was positively associated with increases in WC (P for trend < 0.001). The risk of PD incidence tended to elevate as WC increased (P for trend < 0.001), indicating that the adjusted HRs of PD incidence in the highest WC group versus the reference group was 1.16 (95% CI, 1.10–1.23), whereas it was 0.91 (95% CI 0.84–0.98) in the lowest WC group. Individuals with abdominal obesity were significantly associated with an increased PD risk (HR 1.10, 95% CI: 1.07–1.13). These associations persisted even after adjustment for body mass index and stratification by sex. Even among non-obese individuals, abdominal obesity was associated with a higher PD risk (adjusted HR 1.13, 95% CI: 1.09–1.18). Taken together, higher WC and abdominal obesity were associated with increased PD risk. Even in non-obese individuals, abdominal obesity was associated with an increased PD risk.

## Introduction

Parkinson’s disease (PD) is the second most common neurodegenerative disease following Alzheimer’s disease and affects 6.1 million individuals worldwide, as estimated in 2016^1^. With the increasing aging population, environmental changes, and improved survival of patients with PD, the prevalence of PD is growing fast among all neurologic disorders globally^2,3^. In South Korea, there has been an upward trend in the age-standardized PD incidence (increasing from 13.6 in 2004 to 26.9 per 100,000 person-years in 2013), with approximately 111,311 individuals affected by PD in 2020^3^.

The precise mechanism of neurodegeneration in PD is not yet fully understood, while aging is known to be the primary risk factor of PD^4,5^. Several environmental toxins, behavioral factors, and cardiometabolic parameters have also been reported as key factors affecting dopaminergic neuronal cell death at the substantia nigra through oxidative stress, mitochondrial dysfunction, protein misfolding, and some inflammatory mechanisms^5–7^. Of these, adiposity might be an underlying etiologic factor for age-related neurodegenerative pathologies of PD, since it plays a role in the depletion of striatal dopamine receptor availability^8^. The deleterious effect of general obesity (measured by body mass index [BMI]) on PD risk has been demonstrated in several epidemiologic studies;^9–11^ however, other studies reported opposite results or even null associations^12–18^. Moreover, studies using anthropometric parameters other than BMI are relatively limited^16,19,20^.

In light of abdominal obesity, Chen et al. prospectively examined two large cohorts of men and women in the United States (US), and concluded that a greater waist circumference (WC) was associated with future development of PD among never smokers^20^. Although another US-based cohort study showed null association between WC and PD risk, a European multi-center cohort study in 2019 revealed that female smokers had a 64% increase in the risk of PD development per 10 cm increase in WC^16,19^. Investigations on the association between central adiposity and PD risk have provided a closer look on the mechanism of PD through the perspective of the effect of adiposity-related inflammation on neurodegeneration. WC is known to be a more reliable parameter for abdominal and visceral accumulation of adipose tissue than BMI. However, most of the previous epidemiologic studies on this issue used European or North American data^16,19,20^ and some studies relied on self-measured WC data^16,19^. Although several studies now suggest a potential link between obesity and pathology of neurodegeneration, these few and contradictory findings on WC and PD development prompted us to assess the impact of central adiposity on PD risk. Therefore, we aimed to examine the association between WC, abdominal obesity, and PD risk using the large-scale cohort data of the South Korean population.

## Results

### Baseline characteristics

The proportions of participants according to the five WC categories were as follows: 2.9% (in group of <70 cm in males, <65 cm in females), 27.3% (70–80 cm in males, 65–75 cm in females), 47.2% (80–90 cm in males, 75–85 cm in females), 19.5% (90–100 cm in males, 85–95 cm in females), and 3.1% (≥100 cm in males, ≥95 cm in females), respectively. Table 1 shows the baseline clinical characteristics of the study population according to WC categories. Individuals in the larger WC levels were older (*P* < 0.001), and the distributions of income level and lifestyle habits, such as smoking status, alcohol consumption, and physical activity, were significantly different among WC categories (*P* < 0.001). The mean values of cardiometabolic parameters, such as BMI, systolic and diastolic blood pressures, total cholesterol, low-density lipoprotein cholesterol, and triglycerides were higher in individuals with greater WC categories (*P* < 0.001). Individuals in the larger WC categories were more likely to have comorbidities, such as obesity, hypertension, diabetes mellitus, dyslipidemia, and chronic kidney disease (*P* < 0.001).

**Table 1:** Baseline characteristics of the study population.

Waist circumference (cm)						
	<70 in males,	70–80 in males,	80–90 in males,	90–100 in males,	≥100 in males,	*P*
	<65 in females	65–75 in females	75–85 in females	85–95 in females	≥95 in females	
*N* (%)	200,827 (2.9)	1,888,818 (27.3)	3,270,108 (47.2)	1 349,990 (19.5)	215,903 (3.1)	
Age (years)	51.7 ± 11.1	51.9 ± 10.0	54.5 ± 10.2	57.0 ± 10.6	58.5 ± 11.1	<0.001
Current smoker	40,854 (20.3)	372,424 (19.7)	724,843 (22.2)	278,550 (20.6)	37,548 (17.4)	<0.001
Alcohol drinker	66,236 (33.0)	725,921 (38.4)	1,438,664 (44.0)	567,702 (42.1)	75,530 (35.0)	<0.001
Regular physical activity	33,378 (16.6)	374,319 (19.8)	687,797 (21.0)	263,245 (19.5)	35,843 (16.6)	<0.001
Low income	39,294 (19.6)	352,221 (18.7)	566,489 (17.3)	239,194 (17.7)	40,038 (18.5)	<0.001
Body mass index (kg/m^2^)	19.2 ± 1.9	21.6 ± 1.9	24.1 ± 2.4	26.7 ± 3.4	30.2 ± 3.1	<0.001
Waist circumference (cm)	63.7 ± 4.0	72.6 ± 3.7	82.0 ± 3.8	90.9 ± 3.5	101.1 ± 13.8	<0.001
Systolic BP (mmHg)	116.3 ± 14.9	119.8 ± 14.9	124.9 ± 15.1	128.8 ± 15.3	132.3 ± 15.9	<0.001
Diastolic BP (mmHg)	72.7 ± 9.9	74.6 ± 9.9	77.6 ± 10.0	79.8 ± 10.2	81.5 ± 10.6	<0.001
Fasting glucose (mg/dL)	93.1 ± 20.9	95.4 ± 21.6	100.4 ± 25.8	105.2 ± 29.3	110.5 ± 33.9	<0.001
Total cholesterol (mg/dL)	187.4 ± 37.8	193.9 ± 39.5	200.9 ± 42.7	204.2 ± 44.6	205.7 ± 46.2	<0.001
HDL-C (mg/dL)	63.8 ± 38.9	59.8 ± 34.4	55.1 ± 34.0	52.9 ± 33.7	52.7 ± 31.9	<0.001
LDL-C (mg/dL)	109.9 ± 90.0	116.2 ± 90.3	120.4 ± 79.8	121.3 ± 82.0	122.1 ± 94.7	<0.001
Triglycerides (mg/dL)^a^	79.3 (79.1–79.5)	94.9 (94.8–95.0)	124.1 (124.1–124.2)	144.8 (144.6–144.9)	152.8 (152.4–153.1)	<0.001
Creatinine (mg/dL)	0.96 ± 1.03	1.02 ± 1.19	1.11 ± 1.38	1.07 ± 1.23	1.03 ± 1.11	<0.001
eGFR (mL/min/1.73m^2^)	87.8 ± 32.4	86.5 ± 35.4	84.5 ± 38.5	83.1 ± 38.1	82.1 ± 36.6	<0.001
Obesity	2093 (1.0)	65,797 (3.5)	1,073,121 (32.8)	1,076,437 (79.7)	208,894 (96.8)	<0.001
Hypertension	28,377 (14.1)	377,398 (20.0)	1,121,280 (34.3)	675,372 (50.0)	139,753 (64.7)	<0.001
Diabetes mellitus	7842 (3.9)	112,027 (5.9)	381,229 (11.7)	252,038 (18.7)	59,647 (27.6)	<0.001
Dyslipidemia	18,677 (9.3)	276,529 (14.6)	776,766 (23.8)	430,785 (31.9)	83,969 (38.9)	<0.001
Chronic kidney disease	11,623 (5.8)	119,080 (6.3)	269,459 (8.2)	139,635 (10.3)	28,500 (13.2)	<0.001

Values are presented as numbers (percentages) or means ± standard deviations.

^a^Geometric means (95% confidence intervals).

*BP*, blood pressure; *HDL-C*, high-density lipoprotein cholesterol; *LDL-C*, low-density lipoprotein cholesterol; *eGFR*, estimated glomerular filtration rate.

### Associations of WC and abdominal obesity with PD incidence

**Fig. 1:**
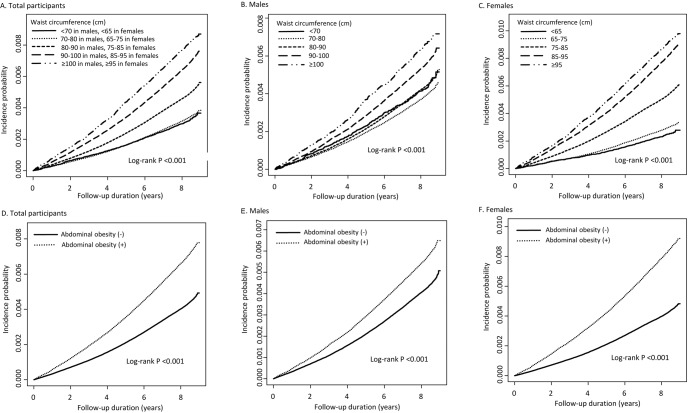
Kaplan–Meier estimates for the probability of incident Parkinson's disease according to waist circumference and the presence of abdominal obesity (all log-rank *P* values < 0.001). The probability of incident Parkinson's disease according to waist circumference was analyzed for total participants (**A**), males (**B**), and females (**C**). Analysis according to the presence or absence of abdominal obesity among total participants (**D**), males (**E**), and females (**F**) were also presented.

A total of 33,300 (0.48%) individuals were diagnosed with PD during the follow-up. Figure 1 shows the Kaplan–Meier curves for incidence probabilities of PD with respect to WC categories and abdominal obesity. The incidence probabilities significantly increased in the higher WC categories in total, male, and female participants (all log-rank P-value < 0.001). The plot showed that the cumulative PD incidence increased in total, male, and female participants with abdominal obesity compared with each group without abdominal obesity (all log-rank P-value < 0.001). Table 2 presents the adjusted hazard ratios (HRs) (95% confidence intervals [CIs]) of PD in five WC categories. Incrementally higher HRs of PD were observed with higher WC categories in the total, male, and female population irrespective of adjustment for confounding variables (all P for trend < 0.001). In total participants, compared with the second highest level of WC (70–80 cm in males and 65–75 cm in females) as the reference group, the smallest WC group was associated with a 9% lower PD risk (HR: 0.91, 95% CI: 0.84–0.98), and the largest WC category was associated with a 16% higher PD risk (1.16, 95% CI: 1.10–1.23). In male and female participants, the adjusted HRs of PD in model 2 were 25% (HR: 1.25, 95% CI: 1.14–1.36) and 15% (1.15, 95% CI: 1.07–1.23) higher in the largest WC group than in the reference groups. These associations remained significant after adjusting for baseline BMI.

**Table 2:** Longitudinal associations between waist circumference categories and incident Parkinson’s disease.

Waist circumference (cm)	*N*	Event	Person-years	Incidence rate^a^	Model 1^b^	Model 2^c^	Model 3^d^
Total participants							
<70 in males, <65 in females	200,827	623	1,619,243	0.38	0.95 (0.87–1.03)	0.91 (0.84–0.98)	0.90 (0.83–0.98)
70–80 in males, 65–75 in females	1,888,818	6267	15,457,638	0.40	1.00 (reference)	1.00 (reference)	1.00 (reference)
80–90 in males, 75–85 in females	3,270,108	15,705	26,754,898	0.58	1.44 (1.40–1.49)	1.10 (1.07–1.13)	1.11 (1.07–1.14)
90–100 in males, 85–95 in females	1,349,990	9018	10,992,717	0.82	2.02 (1.95–2.09)	1.18 (1.14–1.22)	1.20 (1.14–1.24)
≥100 in males, ≥95 in females	215,903	1687	1,742,378	0.96	2.39 (2.26–2.52)	1.16 (1.10–1.23)	1.19 (1.11–1.27)
*P* for trend					<0.001	<0.001	<0.001
Males							
<70	76,845	318	599,779	0.53	1.12 (0.99–1.25)	0.86 (0.76–0.96)	0.84 (0.75–0.95)
70–80	777,830	2981	6,266,622	0.47	1.00 (reference)	1.00 (reference)	1.00 (reference)
80–90	1,806,641	7883	14,675,091	0.53	1.13 (1.08–1.17)	1.10 (1.05–1.15)	1.12 (1.06–1.17)
90–100	735,264	4039	5,947,447	0.67	1.43 (1.36–1.49)	1.18 (1.12–1.24)	1.22 (1.14–1.30)
≥100	92,597	593	740,229	0.80	1.69 (1.55–1.84)	1.25 (1.14–1.36)	1.32 (1.19–1.47)
*P* for trend					<0.001	<0.001	<0.001
Females							
<65	123,982	305	1,019,464	0.29	0.84 (0.75–0.94)	0.95 (0.85–1.07)	0.95 (0.85–1.07)
65–75	1,110,988	3286	9,191,017	0.35	1.00 (reference)	1.00 (reference)	1.00 (reference)
75–85	1,463,467	7822	12,079,808	0.64	1.81 (1.74–1.88)	1.13 (1.08–1.18)	1.13 (1.08–1.18)
85–95	614,726	4979	5,045,270	0.98	2.76 (2.64–2.88)	1.21 (1.16–1.27)	1.21 (1.15–1.29)
≥95	123,306	1094	1,002,149	1.09	3.05 (2.85–3.27)	1.15 (1.07–1.23)	1.15 (1.05–1.26)
*P* for trend					<0.001	<0.001	<0.001

^a^Incidence per 1000 person-years

^b^Model 1: non-adjusted.

^c^Model 2: adjusted for age, sex, smoking status, alcohol consumption, physical activity, income, hypertension, diabetes mellitus, dyslipidemia, and chronic kidney disease.

^d^Model 3: adjusted for variables in model 2 and body mass index

Table 3 shows the adjusted HRs (95% CIs) of PD in the individuals with abdominal obesity compared with that of those without abdominal obesity. After adjusting for potential confounding variables (model 2), the HRs for PD increased significantly among individuals with abdominal obesity compared with those without in total (HR: 1.09, 95% CI: 1.07–1.13), male (1.11, 95% CI: 1.07–1.15), and female participants (1.10, 95% CI: 1.07–1.14). These associations persisted even after further adjusting for BMI (model 3).

**Table 3:** Longitudinal associations between abdominal obesity and incident Parkinson’s disease.

Abdominal obesity	*N*	Event	Person-years	Incidence rate^a^	Model 1^b^	Model 2^c^	Model 3^d^
Total participants							
No	5,359,753	22,595	43,831,779	0.51	1.00 (reference)	1.00 (reference)	1.00 (reference)
Yes	1,565,893	10,705	12,735,095	0.84	1.63 (1.59–1.67)	1.10 (1.07–1.13)	1.09 (1.07–1.12)
* P*-value					<0.001	<0.001	<0.001
Males							
No	2,661,316	11,182	21,541,491	0.51	1.00 (reference)	1.00 (reference)	1.00 (reference)
Yes	827,861	4632	6,687,677	0.69	1.33 (1.29–1.38)	1.11 (1.07–1.15)	1.10 (1.06–1.14)
* P*-value					<0.001	<0.001	<0.001
Females							
No	2,698,437	11,413	22,290,288	0.51	1.00 (reference)	1.00 (reference)	1.00 (reference)
Yes	738,032	6073	6,047,418	1.00	1.96 (1.90–2.02)	1.10 (1.07–1.14)	1.09 (1.06–1.13)
* P*-value					<0.001	<0.001	<0.001

^a^Incidence per 1000 person-years

^b^Model 1: non-adjusted.

^c^Model 2: adjusted for age, sex, smoking status, alcohol consumption, physical activity, income, hypertension, diabetes mellitus, dyslipidemia, and chronic kidney disease.

^d^Model 3: adjusted for variables in model 2 and body mass index

### Subgroup analysis

**Fig. 2:**
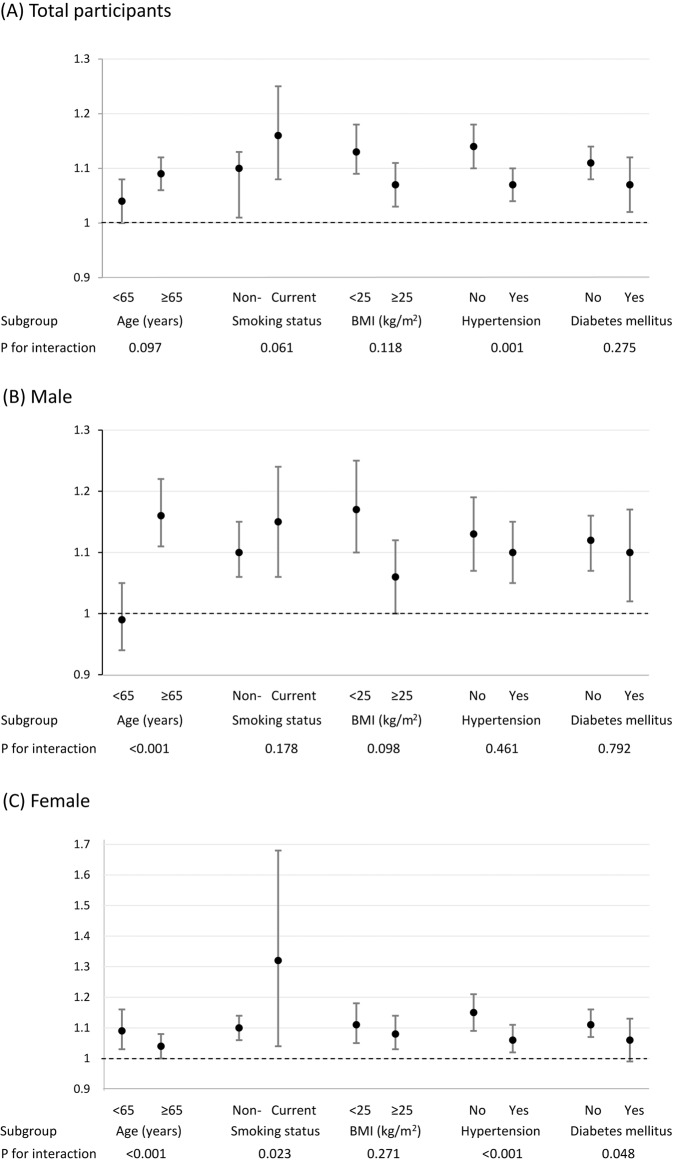
Adjusted hazard ratios (95% confidence intervals) of Parkinson’s disease according to the presence of abdominal obesity in subgroups. Associations between abdominal obesity and the risk of Parkinson's disease in subgroups stratified by age, smoking status, BMI, hypertension, and diabetes mellitus among total participants (**A**), males (**B**), and females (**C**) were analyzed. *P*-values for interaction were calculated using multivariable Cox proportional hazard regression models after adjusting for age, sex, smoking status, alcohol consumption, physical activity, income, hypertension, diabetes mellitus, dyslipidemia, and chronic kidney disease.

Figure 2 shows the associations between abdominal obesity and PD risk in subgroups stratified by age, smoking status, BMI, hypertension, and diabetes mellitus. The associations interacted with age differently between males and females. Positive associations between abdominal obesity and PD risk were stronger among males aged ≥65 years than those aged <65 years, while the associations were stronger among females aged <65 years than those aged ≥65 years. There was a significant interaction with smoking status in the association of abdominal obesity with PD risk in female individuals (P for interaction = 0.023), and the association was stronger in those currently smoking than those who were not. Although increased PD risks in abdominal obesity were observed regardless of the presence of general obesity based on BMI ≥ 25 kg/m^2^, there was no significant interaction with respect to obesity status. The association between abdominal obesity and PD risk was stronger in the total and female populations without hypertension than in those with hypertension (*P* for interaction < 0.001), and the association was stronger in females without diabetes mellitus than in those with diabetes mellitus (*P* for interaction = 0.048).

## Discussion

This large-scale Asian cohort study investigated the association between professionally measured WC and PD risk. In this nationwide cohort, which included 6,925,646 participants aged ≥40 years with 8.35 years of follow-up, we found that higher WC and the presence of abdominal obesity was significantly associated with an elevated PD risk both in males and females. Notably, abdominal obesity was associated with an increased PD risk even in individuals without general obesity. Each subgroup based on age, smoking status, and comorbidities showed consistent results that abdominal obesity was associated with an increased PD risk, although the level of interaction was different according to sex.

Only a few studies have reported the association between WC and PD risk, with inconsistent findings. One recent large-scale European cohort study and another US health professional cohort study revealed a statistically significant positive association between WC and PD risk among women^19,20^. Furthermore, WC was positively associated with PD risk after multivariable adjustment in a nationwide cohort study examining metabolic syndrome and PD risk^21^. In contrast, a few population-based studies showed a null association between WC and PD risk^16,17^. Consistently with our findings, the Honolulu-Asia aging study has also reported that high subscapular skinfold thickness of participants was positively associated with PD risk, whereas the leanest participants were found to have the lowest PD incidence over 30 years of follow-up^9^.

Although pathophysiology under the association between central obesity and subsequent PD development as shown in our study and several prior studies has not been fully revealed, insulin resistance appears to act as a key modulator in the link between them. A strong link between obesity induced insulin resistance and neurodegeneration through brain insulin dysregulation has been demonstrated previously^22,23^. Central insulin effects are considered as neurotrophic, and insulin signaling impairment plays an important role in increasing intracellular protein aggregates, mainly alpha-synuclein protein in the substantia nigra^22–24^. In accordance with dysregulated insulin signaling, mitochondrial dysfunction in the brain is involved in metabolic disturbance and neurodegeneration^25,26^. A previous epidemiologic finding showed that metabolic syndrome, a cluster of abdominal obesity and insulin resistance, is associated with the PD risk^21^. In addition, obesity is associated with chronic inflammation states, which is characterized by elevated production of proinflammatory cytokines, thereby leading to oxidative stress and neuronal death^26,27^. Also, recent evidence on the effect of gut microbiota on α-synucleinopathy or neuroinflammation suggests a dysbiosis-mediated link between adiposity and PD pathogenesis, based on an established association between adiposity and altered gut microbiota^28^.

Besides insulin resistance, accumulated visceral adipose tissue acts as an endocrine organ secreting adipokines, such as leptin and adiponectin, which play an important role in neurodegeneration^29–31^. Although leptin primarily controls appetite via acting on hypothalamic regions, leptin receptors are expressed in extra-hypothalamic regions, including the substantia nigra. Leptin has a protective effect on dopaminergic neuron degeneration by mediating intracellular signaling via the JAK-STAT and ERK/CREB pathways, thus preserving the function of the dopamine system^29,31,32^. The level of adipokine is inversely associated with central fat distribution^33^, and individuals with abdominal obesity are suspected to have vulnerability in neuroprotection against 6-hydroxydopamine toxicity, which can accelerate dopaminergic neuron degeneration^29,31^.

Above all, even normal-weight individuals with abdominal obesity showed an increased PD risk in our results. While normal-weight central obesity and its associated negative health outcomes have been widely investigated^33,34^. Our results imply that neurodegenerative diseases, including PD, can be one of those poor health outcomes of abdominal obesity even in non-obese individuals through the deleterious effects of visceral adipose tissue.

Subgroup analysis in our study showed some interesting results. There was a prominently positive association between abdominal obesity and PD risk in female current smokers and the risk was 1.32-fold higher in female current smokers with abdominal obesity than in those without abdominal obesity. A few studies have investigated the relationship between central adiposity and PD risk according to smoking status, although the findings differed^16,19,20^. Chen et al. reported that the higher WC was associated with an increased PD risk among never smokers of both sexes and female ever smokers^20^. Another evidence has highlighted the smoking status as an effect modifier of PD risk in female smokers^19^; however, Palacios et al. did not observe any significant associations^16^. Current evidence in the genetics of PD may partly explain these contrasting findings, suggesting that ethnicity-specific mutations or variants may have different contributions to the etiologies of PD development^3,35^. The combination of smoking status and visceral fat distribution might have a different influence on PD risk depending on ethnicity as well as genetic factors^36,37^. Furthermore, epidemiologic characteristics of the Asian-Pacific region indicate a distinct female predominance of PD prevalence and low smoking rates among females^3^, which could also modify our findings that the PD risk among female smokers with abdominal obesity was more prominent than that among not currently smoking females. This interpretation could be applicable to our findings that males and females showed different interactions with age in the association between abdominal obesity and PD risk. Meanwhile, it is noteworthy that abdominal obesity increased the PD risk irrespective of the presence of diabetes mellitus in our findings, given that diabetes is known as one of risk factors of PD through shared genetic predisposition and pathophysiologic mechanisms^38^. Furthermore, abdominally obese females without diabetes mellitus or hypertension had an increased PD risk compared with abdominally nonobese females without the disease, indicating the possible role of abdominal obesity in PD development in these subgroups.

We acknowledge that some potential limitations should be considered when interpreting the current data. First, a reverse causality might exist between WC and PD risk because of the retrospective cohort design, although we considered a 1-year lag period in identifying PD to overcome this issue. Second, the operational diagnosis of PD based on claim database may cause a possibility of misdiagnosis. However, our diagnostic criteria using the rare and incurable disease registry in the NHIS database could identify PD cases more reliably. Third, the ethnic homogeneity of our data limits the generalizability of our study findings; thus, further studies will be needed in Asian populations with different ethnic backgrounds as well as other races. Fourth, due to the lack of information on the NHIS database, we could not adjust for some confounders affecting WC and PD incidence, such as nutritional intake, including coffee and caffeine intake, education, occupation, and history of exposure to toxic molecules including pesticides. Despite these limitations, our study has several strengths. This study determined the relationship between abdominal obesity and PD risk using the largest cohort database in Asia. The ethnic homogeneity of our study could be viewed as a strength since important differences in genetic factors, etiologies, and management of PD according to the regions are emerging. We confirmed the associations using very large-scale cohort data of South Koreans, which enabled us to consider various confounding variables and detailed subgroup analyses. Another strength of this study was the relatively long-term follow-up period of 8.3 years, which allowed us to more truly examine the causality of abdominal obesity and PD risk. Additionally, we had the WC measured directly by trained health professionals instead of using self-reported data.

In conclusion, in this large, nationwide cohort study comprising South Korean individuals aged ≥40 years, we found a positive association of WC and abdominal obesity with PD risk. WC represents central fat distribution and the level of visceral fat level more accurately than BMI, which makes WC a good indicator of insulin resistance. Our findings suggest that a higher WC and abdominal obesity reflect a PD risk in both obese and non-obese individuals. Of note, our findings can add clarity to the current evidence on the role of abdominal obesity in PD risk and provide valuable insights to risk reduction interventions of PD. Future research is needed to investigate the potential roles of visceral fat-driven adipokines and insulin resistance on PD and neurodegeneration.

## Methods

### Data sources

South Korea has launched a single, mandatory universal health insurance system called the National Health Insurance Service (NHIS) in 2000, which almost covers the entire South Korean population. The NHIS in South Korea contains electronic records for sociodemographic variables; health care utilization-related data for all insured people with disease diagnosis data based on the International Classification of Diseases, 10th Revision, Clinical Modification (ICD-10-CM); and mortality data accumulated since 2002. Furthermore, the information on health screening results obtained through the free mandatory National Health Screening Program at least biennially for adults aged ≥19 years are included in the NHIS database. An individual researcher can use the NHIS database under permission granted from the NHIS with the appropriate research proposal for the use of data. The protocol for this study was approved by the NHIS review committee and Institutional Review Board of Korea University Anam Hospital in accordance with the Declaration of Helsinki of the World Medical Association (IRB No. 2019AN0201). The requirement for informed consent was waived as all data used in the analysis was anonymized and de-identified according to strict confidentiality guidelines.

### Study participants

Of the 10,585,844 individuals who underwent the national health screening offered by the NHIS between January 1 and December 31 in 2009, we excluded 3,345,043 individuals aged <40 years, 274,452 with missing values for the analysis, and 12,786 diagnosed with PD between 2002 and study enrollment. We further excluded 27,917 individuals diagnosed with PD within 1 year after enrollment. Finally, a total of 6,925,646 individuals were included in the analysis.

### Study outcome and follow-up

Newly diagnosed PD was identified on the basis of the ICD-10-CM code for PD (G20), with the PD registration code (V124) in the rare and incurable diseases registry in the NHIS database between January 2010 and December 2018. The rare and incurable disease registry, which also includes PD, has been implemented by the South Korean government since 2006 for copayment reduction by providing financial support to reduce patients’ medical expenses burden. Study participants were monitored until the date of PD diagnosis, date of death, or December 31, 2018, whichever came first. The median follow-up duration for the study endpoint was 8.35 years (interquartile range 8.11–8.60).

### Definition of abdominal obesity and WC categories

WC was measured at the narrowest point between the inferior border of the rib cage and iliac crest during minimal respiration. The cutoff point for WC for abdominal obesity in South Koreans was defined as 90 cm for males and 85 cm for females^39^. WC was categorized into five levels as follows: <70, 70–80, 80–90, 90–100, and ≥100 cm in males; <65, 65–75, 75–85, 85–95, ≥95 cm in females.

### Other variables

The data for each participant’s smoking status, alcohol consumption, and physical activity were obtained by self-report questionnaire. Smoking status was classified into current smokers or nonsmokers based on the participants’ smoking history. Current smokers were defined as individuals who answered they are currently smoking, and nonsmokers as those who had never smoked or had quit smoking. Participants who had consumed any amount of alcohol were defined as alcohol drinkers. Regular exercise was defined as working out, including either ≥30 min of moderate physical activity at least five times per week or ≥20 min of vigorous physical activity at least three times per week. Income level was dichotomized at the lower 25%, based on employee health insurance premiums reflecting a worker’s salary. BMI was calculated as a participant’s weight in kilograms divided by the square of their height in meters. Obesity was defined as BMI ≥ 25 kg/m^2^ based on the World Health Organization recommendations for Asian populations^40^. Systolic and diastolic blood pressure was measured using a standard mercury sphygmomanometer. Serum glucose, lipid, and creatinine levels were measured after an overnight fast. These health examinations were performed in hospitals certified by the NHIS under regular quality control.

The presence of comorbidities, such as hypertension, diabetes mellitus, dyslipidemia, and chronic kidney disease was defined based on the combination of health examination results and ICD-10-CM codes. Hypertension was defined as (a) systolic/diastolic blood pressure ≥140/90 mmHg or (b) having one or more claims for prescription of antihypertensive medications per year under ICD-10-CM codes I10–13 and I15. Diabetes mellitus was defined as (a) serum fasting glucose level ≥126 mg/dL or (b) having one or more claims for prescription of antidiabetic medication per year under ICD-10-CM code E10–14. Dyslipidemia was defined as (a) serum total cholesterol level ≥240 mg/dL or (b) having one or more claims for prescription of lipid-lowering agents per year under ICD-10-CM code E78. Chronic kidney disease was defined as estimated glomerular filtration rate <60 mL/min/1.73 m^2^ using the Modification of Diet in Renal Disease equation^41^.

### Statistical analyses

Baseline characteristics are presented as means ± standard deviations for continuous variables and numbers (percentages) for categorical variables according to baseline WC categories. One-way analysis of variance for continuous variables and Pearson’s chi-squared test for categorical variables were used to compare baseline characteristics among the five WC groups. The PD incidence rate was calculated as event number per 1,000 person-years. The cumulative PD incidence according to the five WC categories and the presence or absence of abdominal obesity was plotted using the Kaplan–Meier curves, and the log-rank test was performed to analyze differences among the groups. To investigate the association between WC, abdominal obesity, and PD risk, HRs and 95% CIs values were calculated using the multivariable Cox proportional hazards regression models. Model 1 was not adjusted. Model 2 was adjusted for age, sex, smoking status, alcohol consumption, physical activity, income, hypertension, diabetes mellitus, dyslipidemia, and chronic kidney disease. We further adjusted for baseline BMI in model 3 in addition to the variables adjusted in model 2. These analyses were also performed after stratifying by sex. We performed subgroup analyses by age, smoking status, baseline BMI, hypertension, and diabetes mellitus to investigate the associations between abdominal obesity and PD development in these subgroups. P-value for interaction was calculated using Cox regression analyses. Statistical analyses were performed using SAS version 9.4 (SAS Institute Inc., Cary, NC, USA). Statistical significance was set at *P* < 0.05.

## Data Availability

This study was performed using the National Health Insurance System database (https://nhiss.nhis.or.kr/), and the results do not necessarily represent the opinion of the National Health Insurance Corporation. Restrictions apply to the availability of these data, which were used under license for this study.
